# Incidence and predictors of attrition among patients receiving ART in eastern Zimbabwe before, and after the introduction of universal ‘treat-all’ policies: A competing risk analysis

**DOI:** 10.1371/journal.pgph.0000006

**Published:** 2021-10-13

**Authors:** Malebogo Tlhajoane, Freedom Dzamatira, Noah Kadzura, Constance Nyamukapa, Jeffrey W. Eaton, Simon Gregson

**Affiliations:** 1 Department for Infectious Disease Epidemiology, Imperial College London, London, United Kingdom; 2 Department of Population Health, London School of Hygiene and Tropical Medicine, London, United Kingdom; 3 Biomedical Research and Training Institute, Harare, Zimbabwe; Columbia University - MSPH, ZIMBABWE

## Abstract

As HIV treatment is expanded, attention is focused on minimizing attrition from care. We evaluated the impact of treat-all policies on the incidence and determinants of attrition amongst clients receiving ART in eastern Zimbabwe. Data were retrospectively collected from the medical records of adult patients (aged≥18 years) enrolled into care from July 2015 to June 2016—pre-treat-all era, and July 2016 to June 2017—treat-all era, selected from 12 purposively sampled health facilities. Attrition was defined as an absence from care >90 days following ART initiation. Survival-time methods were used to derive incidence rates (IRs), and competing risk regression used in bivariate and multivariable modelling. In total, 829 patients had newly initiated ART and were included in the analysis (pre-treat-all 30.6%; treat-all 69.4%). Incidence of attrition (per 1000 person-days) increased between the two time periods (pre-treat-all IR = 1.18 (95%CI: 0.90–1.56) *versus* treat-all period IR = 1.62 (95%CI: 1.37–1.91)). In crude analysis, patients at increased risk of attrition were those enrolled into care during the treat-all period, <34 years of age, WHO stage I at enrolment, and had initiated ART on the same day as HIV diagnosis. After accounting for mediating clinical characteristics, the difference in attrition between the pre-treat-all, and treat-all periods ceased to be statistically significant. In a full multivariable model, attrition was significantly higher amongst same-day ART initiates (aSHR = 1.47, 95%CI:1.05–2.06). Implementation of treat-all policies was associated with an increased incidence of ART attrition, driven largely by ART initiation on the same day as HIV diagnosis which increased significantly in the treat all period. Differentiated adherence counselling for patients at increased risk of attrition, and improved access to clinical monitoring may improve retention in care.

## 1 Introduction

Increasing empirical evidence has shown the clinical benefits of antiretroviral therapy (ART) for all people living with HIV (PLHIV) regardless of their immunological status [1, 2]. These include a reduction in rates of severe HIV-related illness and mortality, approximately 23% reported in Uganda and Kenya, improved levels of viral suppression at the population level, and a reduction in HIV incidence [3–5]. In Botswana, Zambia and South Africa for example, reductions of 20% to 30% in HIV incidence were observed in the intervention arms of two clinical trials when compared to the control arms [4, 6, 7]. These findings prompted the World Health Organization (WHO) to recommend immediate initiation of ART to all PLHIV through a policy initiative known as ‘treat-all’ [8]. As of July 2019, all countries in the WHO eastern and southern Africa region had adopted the treat-all strategy and 72% of PLHIV in the area were receiving ART, an increase from 54% in 2015 [9, 10].

While the benefits of treat-all have been well documented, for these to be fully optimized, PLHIV need to be continuously engaged in care and adherent to ART. Attrition (non-retention in care), however, has been described at all stages of the HIV treatment cascade, threatening the success of national treatment programmes [11]. Several studies have documented high rates of attrition (≥10%) amongst those receiving ART [12–15], a phenomenon that is of particular interest as treat-all policies are scaled-up, and more PLHIV are initiated onto treatment. This is of concern particularly amongst those with higher CD4 cell counts (>500 cells/mm^3^) who are now eligible for ART and may be more likely to experience attrition [16]. Early evidence has indicated comparable rates of attrition between pre-treat all and—treat-all era ART initiates in Rwanda and Eswatini [17, 18], although attrition was significantly higher in crude analysis amongst patients initiated under treat-all conditions in Eswatini compared to standard of care. In Nigeria, 34% of newly initiated patients were reported to be lost from care, in the treat all cohort, compared to 19% in the pre-treat all group [19]. Similar findings were reported in Zimbabwe by Makurumidze and colleagues [20]. In this study however, patient-level data were collected from a national electronic patient monitoring system (ePMS), more commonly used in high-volume health facilities which may differ from smaller primary care facilities in service provision [20].

The disparate nature of these findings indicates a need for additional programmatic evidence to better understand treatment outcomes and the nature of loss-to-follow-up in high-burden countries. In addition, questions remain on how determinants of attrition may be altered by the introduction of treat-all guidelines, particularly patient characteristics such as CD4 cell count and WHO clinical staging. Previously, correlates of attrition have been same-day ART initiation, young age, sex, and being unmarried [13, 18, 21–23]. Facility-level factors have also been noted. For example, levels of attrition were found to be lower, in rural Mozambique and Uganda, in areas where community ART groups were used, and higher, in Malawi, in hospitals compared to smaller, more decentralised health centres [24–26].

Amongst high-burden countries in sub-Saharan Africa, Zimbabwe was one of the first to adopt treat-all recommendations [27]. In July 2016, a pilot programme began to guide the implementation of treat-all policies nationally, with recommendations formally adopted in December 2016 [28]. In this study, we evaluate the effects of the introduction of treat-all guidelines on treatment outcomes, including the incidence and predictors of attrition following ART initiation, within routine HIV programme settings in the Manicaland province of Zimbabwe.

## 2 Methods

### 2.1 Study design and data sources

A retrospective cohort study was conducted within 12 purposively sampled health facilities in two predominantly rural districts—Mutasa and Makoni—in Manicaland province, east Zimbabwe. These districts included the research sites for a longitudinal population-based survey (1998–2013) and were selected by the Government of Zimbabwe to pilot the implementation of treat-all beginning in July 2016 [27, 29]. Health facilities were equally and purposively selected from the two study districts to include eight primary-care facilities (large health centres (LHCs) and small clinics (SCs)), and four first referral level facilities (two district hospitals and two rural hospitals). Selection was done from a sampling frame of all public health facilities in each district (n = 91). Descriptive characteristics of the health facilities included in the study are provided in S1 Table.

Routine clinical data were extracted from the medical records of all patients aged ≥18 years on the date of enrolment into care. Data extraction occurred between August and September 2017. Inclusion was limited to patients enrolled and initiated on ART between July 2015 and June 2017. In addition to the patient’s individual medical record, data were extracted from clinic registers and electronic patient monitoring systems where available. All data extracted from clinical records were anonymised prior to extraction.

### 2.2 Data management and definition of outcomes

**Fig 1:**
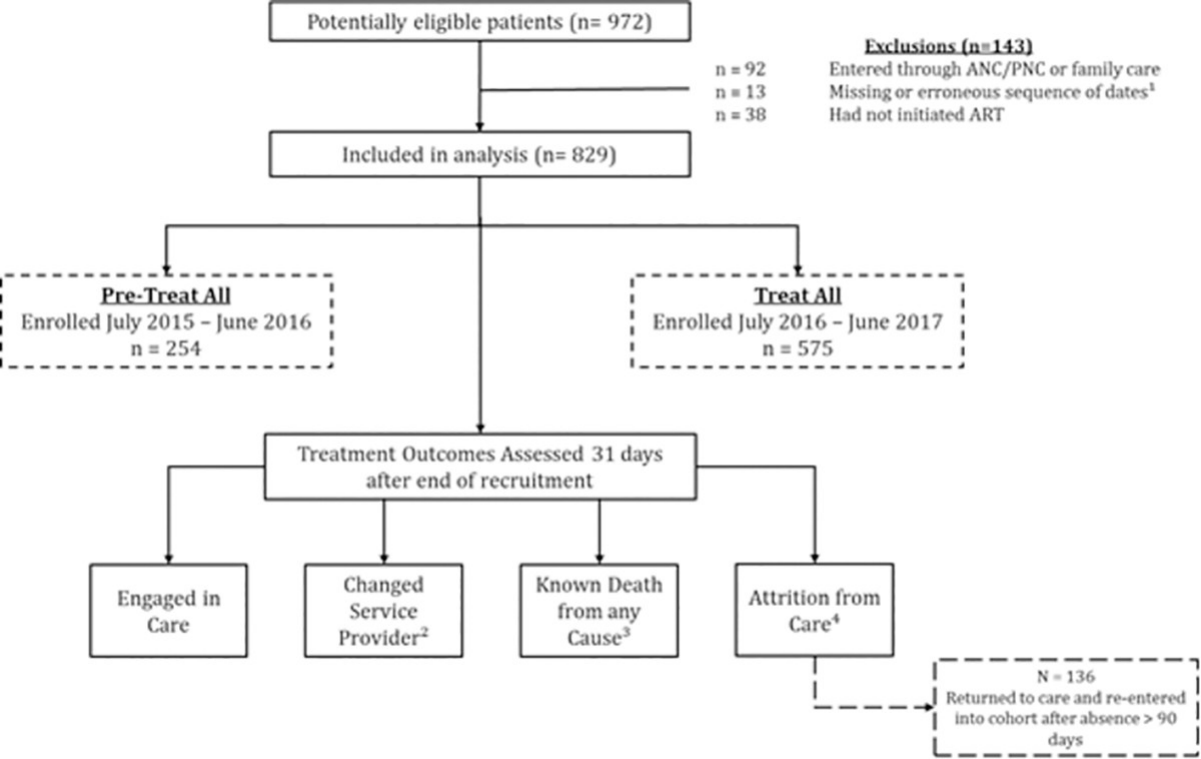
Study flow and treatment outcomes under analysis. ^1^ Missing treatment initiation date, sequence of dates erroneous and could not be determined reliably, or patients who initiated ART after the follow-up time period. ^2^ Transferred to health facility outside of study cohort. ^3^ Mortality from any cause recorded in the medical record. ^4^ Absence from care greater than 90 days.

The pre- treat-all cohort was formed of patients who were enrolled into care from July 2015 to June 2016 while the treat-all cohort included patients enrolled into care between July 2016 and June 2017. Where the date of linkage into care was missing, the ART initiation date was used to determine cohort group (n = 44). Patients who entered into pre-ART care through the family planning programme (i.e. antenatal or postnatal services) were omitted from further analysis given the difference in services provided to those attending family care (n = 92). Treatment outcomes were assessed among patients who had initiated ART (n = 829). Records with a missing treatment initiation date, where the sequence of dates was erroneous and could not be determined reliably, or for patients who initiated ART after the follow-up time period (n = 13) were omitted (Fig 1).

Fig 1 illustrates the study flow and exclusion criteria, as well the treatment outcomes under analysis. Four categories of treatment outcome following ART initiation were defined: (i) engaged in care; (ii) changed service provider (CSP); (iii) death; and (iv) attrition from care (AFC). Those who had CSP to facilities outside of the study cohort were censored on the day of their last clinic visit. Clients who had transferred between facilities included in the study were not considered to have CSP. Death was defined as a known death from any cause reported within a medical record. Where a date of death was not reported, the date of the last clinic appointment was used instead. AFC was defined as an absence of more than 90 days since the last recorded clinic appointment. Amongst those who experienced AFC, survival time was calculated from the date of ART initiation to 90 days after the last clinic appointment. Those who returned into care following an absence of more than 90 days were re-entered into the cohort and treated as continuously engaged (n = 136). A sensitivity analysis was conducted to determine incidence of AFC where these individuals are not re-introduced into the cohort (S2 Table). Analyses were limited to patients who could be followed for at least 90 days. Outcomes were evaluated at 31 days following the end of recruitment for each study period (i.e. 31 July 2016 for pre- treat-all and 31 July 2017 for treat-all), ensuring an equal follow-up time for each study cohort. Where survival time was equal to zero days, this was altered to one.

Where age at clinic enrolment was missing, this was calculated using the patient’s date of birth and date of enrolment into care. A binary variable was created to assess the availability of a baseline CD4 count measure. Where possible, the CD4 count was recorded numerically and a four-level categorical variable was created to evaluate the proportions at each level (0–200 cells/mm^3^; 201–350 cells/mm^3^; 351–500 cells/mm^3^; and ≥501 cells/mm^3^). A baseline CD4 measure was derived using the CD4 count at either programme enrolment or during pre-ART visits if reported within 6 months of ART initiation; at ART initiation; or within 1 month from the ART initiation date if no pre-ART CD4 measure was provided. Educational attainment was collapsed into three groups: (i) none or primary, (ii) secondary or tertiary, and (iii) missing. Timelines were calculated by using clinic visit dates to determine the number of days between HIV diagnosis and ART initiation. Numeric values were retained and a binary variable was created to evaluate the proportion of clients initiated onto ART on the same day as HIV diagnosis. A four-level categorical variable was also created (same day, 1–7 days, 8–28 days, and >28 days). WHO stages III and IV were combined due to the small number of patients in group IV. Variables of interest were assessed for missing observations and categorized as missing where appropriate.

### 2.3 Data analysis

Baseline socio-demographic characteristics and treatment outcomes were compared for the pre-treat all and treat-all periods. The Pearson chi-squared test was used to evaluate differences in proportions between two groups. Incidence of AFC was estimated using person-days and calculated for treatment time period and for all variables under analysis. A plot of the cumulative incidence function was produced to compare the probability of attrition in the pre-treat all and treat all time periods, in the absence of competing events. Logrank p-values were used to test the hypothesis of equal failures. Competing risk regression methods were used to estimate sub-hazard ratios (SHRs) and corresponding 95% confidence intervals (95%CIs) and p-values [30]. These were selected to account for death as a competing event, distinct from censored observations. Robust standard errors were used to account for facility-level correlation.

Covariates were determined *a priori* from published literature with the addition of study district and health facility variables (health facility type and managing authority). Categorical predictor variables were analysed as factor variables to generate measures of effect for each level or group. Regression models were fitted in five stages. Bivariate analysis was conducted, followed by three multivariable models adjusting separately for: (i) socio-demographic characteristics; (ii) clinical characteristics; and (iii) health facility characteristics. All variables with a crude p-value ≤0.2 were included in a final multivariable model. All data management and statistical analyses were performed using STATA 16 (StataCorp, College Station, Texas, USA).

### 2.4 Ethics approval and consent to participate

Ethical approval was granted by the Imperial College Research Ethics Committee (Ref 16IC3597), the Medical Research Council of Zimbabwe (MRCZ/A/2151), and the Biomedical Research and Training Institute’s Internal Review Board. Patient consent was waived due to the retrospective nature of the study and taking into account that all data were anonymised at the point of extraction.

## 3 Results

### 3.1 Cohort characteristics

A total of 829 patients were newly initiated onto ART between July 2015 and June 2017 and were included in this analysis. Among them, 254 (30.6%) enrolled into care during the pre- treat-all period and 575 (69.4%) during the treat-all period. Table 1 displays the descriptive characteristics of the patient population. Most clients were female; however, the proportion of men increased from 41.1% in the pre- treat-all period to 48.2% in the treat-all period. The proportion of patients who had a CD4 count reported at ART initiation declined from 71.7% in the pre- treat-all period to 18.3% in the treat-all period (p<0.001). Amongst those with a baseline CD4 count recorded, the proportion who initiated ART at a CD4≤200 decreased by 25.5 percentage points. Similarly, the proportions at WHO stage 1 at ART initiation and initiating ART on the same day as HIV diagnosis increased between the two study periods (p<0.001). The median number of days between HIV diagnosis and ART initiation decreased by 21 days (pre- treat-all = 26 days, IQR = 12.5–105; treat-all 5 days, IQR = 0–15).

**Table 1:** Baseline characteristics of the ART patient populations in the pre- treat-all and treat-all periods, Manicaland, Zimbabwe, July 2015 to June 2017.

		Pre- Treat-all	Treat-all	p-value
		n = 254 (30.6%)	n = 575 (69.4%)	
Socio-demographic Characteristics				
Sex				0.061
	Male	104 (41.1%)	276 (48.2%)	
	Female	149 (58.9%)	297 (51.8%)	
Age at Enrolment				0.651
	Mean (SD) Median	38.3 (11.8) 36.5	37.0 (11.3) 35	
	≤34	110 (43.3%)	269 (46.8%)	
	35–44	74 (29.1%)	169 (29.4%)	
	45–54	46 (18.1%)	86 (15.0%)	
	≥55	24 (9.5%)	51 (8.9%)	
Marital Status				0.176
	Married	154 (60.6%)	345 (60.0%)	
	Single	28 (11.0%)	77 (13.4%)	
	Widowed	43 (16.9%)	69 (12.0%)	
	Divorced	26 (10.2%)	68 (11.8%)	
	Missing	3 (1.2%)	16 (2.8%)	
Level of Education				0.590
	None or Primary	60 (23.6%)	131 (22.8%)	
	Secondary or Tertiary	132 (52.0%)	284 (49.4%)	
	Missing	62 (24.4%)	160 (27.8%)	
Clinical Characteristics				
CD4 Count at ART Initiation				
	Baseline CD4 recorded	182 (71.7%)	105 (18.3%)	<0.001
	Mean (SD) Median	264.3 (201.8) 229.5	277.0 (236.9) 220	
	0–200	85 (33.5%)	46 (8.0%)	
	201–350	44 (17.3%)	28 (4.9%)	
	351–500	34 (13.4%)	12 (2.1%)	
	≥501	19 (7.5%)	19 (3.3%)	
	Not recorded	72 (28.4%)	470 (81.7%)	
WHO Stage at ART Initiation				<0.001
	I	34 (13.4%)	242 (42.1%)	
	II	106 (41.7%)	191 (33.2%)	
	III & IV	101 (39.8%)	129 (22.4%)	
	Missing	13 (5.1%)	13 (2.3%)	
Days between HIV diagnosis and ART initiation				<0.001
	Mean (SD) Median	121.8 (326.9) 26	25.3 (92.1) 5	
	Same Day	19 (7.5%)	148 (25.7%)	
	1–7	25 (9.8%)	184 (32.0%)	
	8–28	74 (29.1%)	119 (20.7%)	
	>28	117 (46.1%)	85 (14.8%)	
	Missing	19 (7.5%)	39 (6.8%)	
Health Facility Characteristics				
Health Facility Management				0.683
	Central Government	159 (62.6%)	368 (64.0%)	
	Rural District Council	51 (20.1%)	121 (21.0%)	
	Faith-based Mission	44 (17.3%)	86 (15.0%)	
Study District				0.025
	Mutasa	87 (34.3%)	153 (26.6%)	
	Makoni	167 (65.8%)	422 (73.4%)	
Health Facility Type				0.028
	Hospital	165 (65.0%)	392 (68.2%)	
	Large Health Centre	57 (22.4%)	143 (24.9%)	
	Small Clinic	32 (12.6%)	40 (7.0%)	

### 3.2 Treatment outcomes

Among 829 patients included in the analysis, 803 (96.9%) had complete data on ART initiation and visit dates, and 691/803 (86.1%) had at least 90 days of follow-up after ART initiation. Between the two study periods, a slight increase was observed in the proportion of patients retained in care at the end of follow-up (pre- treat-all: 54.2%; treat-all: 59.0%), however, this result was not statistically significant (Table 2). A decline was observed in the proportion of deaths (pre- treat-all: 9.3%; treat-all: 3.6%; p = 0.002). Transfers to facilities outside the study sites declined (p = 0.042) and cases of attrition from care increased (pre- treat-all: 23.2%; treat-all: 29.1%; p = 0.11).

**Table 2:** Treatment outcomes of patients following ART initiation in the pre- treat-all and treat-all periods, Manicaland, Zimbabwe, July 2015 to June 2017.

	Pre-Treat All	Treat All	p-value
Engaged in Care	117 (54.2%)	280 (59.0%)	0.239
Died	20 (9.3%)	17 (3.6%)	0.002
Changed Service Provider	29 (13.4%)	40 (8.4%)	0.042
Lost from Care	50 (23.2%)	138 (29.1%)	0.106
Total	216	475	0.002

### 3.3 Incidence of attrition

**Fig 2:**
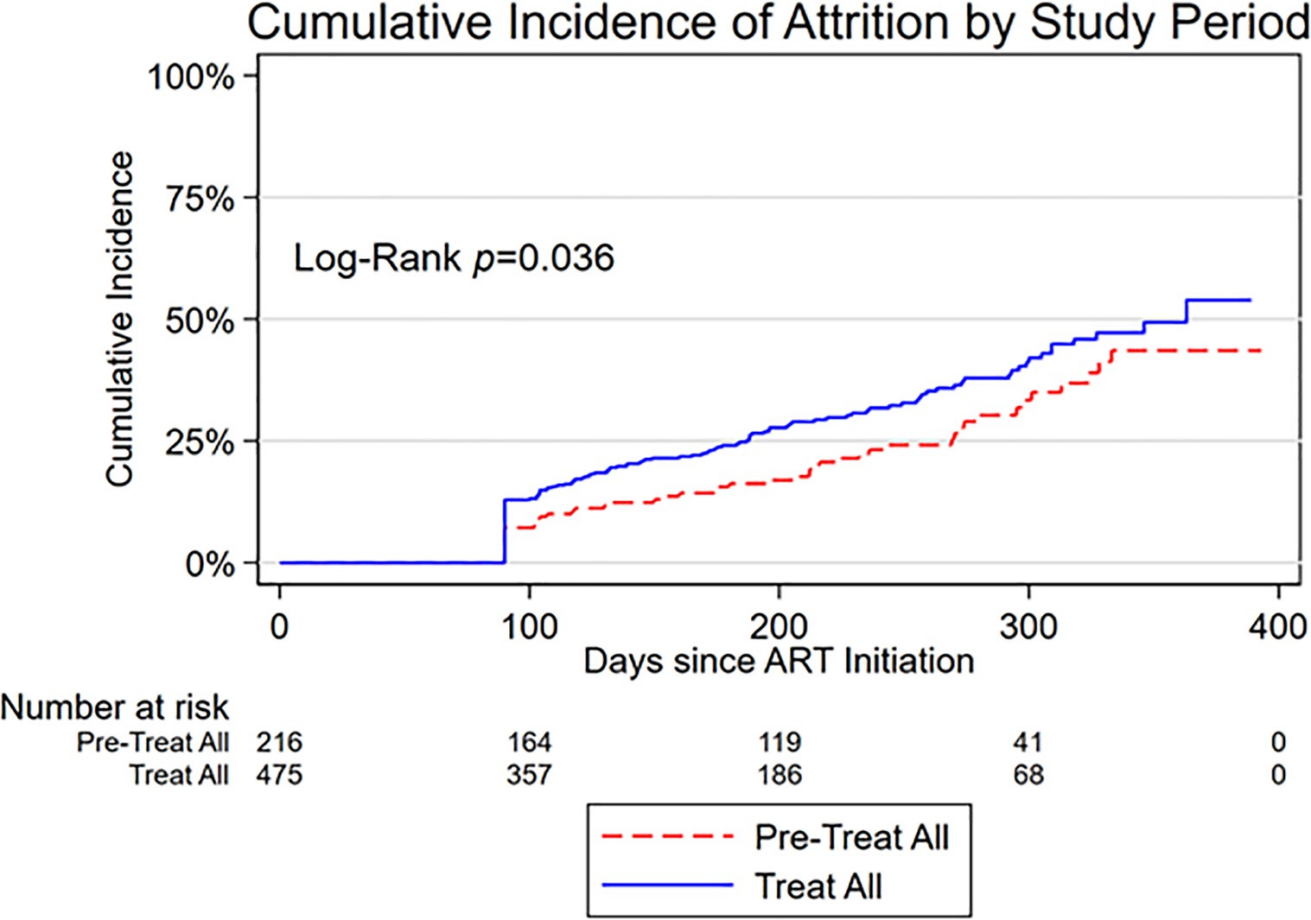
Cumulative incidence curve of attrition from HIV care by time period of enrolment into care (pre- treat-all *versus* treat-all) amongst adult patients following ART initiation.

Overall, the total follow-up time accumulated was 127,831 person days with an incidence of attrition (IR) of 1.47 per 1000 person-days (95%CI: 1.27–1.70). The incidence of attrition was higher during the treat-all period (IR = 1.62; 95%CI: 1.37–1.91) than in the pre- treat-all period (IR = 1.18; 95%CI: 0.90–1.56). Overall, the cumulative incidence of attrition following ART initiation for the combined cohort was 10.8% (95%CI: 8.6–13.4) at 100 days; 24.1% at (95%CI: 20.8–28.0) at 200 days; 37.2% (95%CI: 32.6–42.2) at 300 days, and 50.6% (95%CI: 43.4–58.3) at 400 days. The cumulative incidence of attrition was moderately higher in the treat-all cohort than in the pre- treat-all cohort (log rank p-value = 0.036) (Fig 2), with the difference largest in the early period after ART initiation (approximately 90–300 days).

### 3.4 Determinants of attrition

In the bivariate analysis, treat-all enrolment into care was a predictor of attrition (cSHR = 1.48, 95%CI: 1.09–2.03). This difference ceased to be statistically significant following adjustment for age and clinical characteristics, including CD4 count, same-day ART initiation, and WHO clinical stage at ART initiation (Table 3, Model 2), suggesting that clinical factors may mediate the effect of treatment time period on attrition from care given the observed differences in clinical characteristics between the two time periods (Table 1), as well as the associations between clinical characteristics and study time period (S3 Table). Amongst the clinical factors included in the analysis, having a baseline CD4 cell count recorded, WHO clinical stage at ART initiation, and ART initiation on the same day as HIV diagnosis showed statistically significant associations with attrition (Table 3). The sub-hazard ratio of attrition was lower amongst those who had a CD4 cell count recorded at baseline (cSHR = 0.63, 95%CI: 0.46–0.85) and in those in the later stages of HIV infection (stages II, III and IV *versus* stage I: cSHR = 0.60, 95%CI: 0.45–0.80). These associations ceased to be statistically significant in the multivariable models (Table 3). However, the association between ART initiation on the same day as HIV diagnosis and higher AFC remained statistically significant in the final multivariable model (aSHR = 1.47, 95%CI: 1.05–2.06), with a lower hazard of attrition when the time between HIV diagnosis and HIV initiation was greater than 8 days (Table 3, Model 4).

**Table 3:** Crude and adjusted measures of association between hypothesized predictors and attrition from care.

				Bivariate Analysis	Multivariable Model 1^2^	Multivariable Model 2^3^	Multivariable Model 3^4^	Multivariable Model 4^5^
		Person-days^1^	Incidence Rate (95% CI)	cSHR (95% CI)	aSHR (95% CI)	aSHR (95% CI)	aSHR (95% CI)	aSHR (95% CI)
Enrolment Time Period								
	Pre- Treat-All	42.3	1.18 (0.90–1.56)	1 (Ref)	1 (Ref)	1 (Ref)	1 (Ref)	1 (Ref)
	Treat-All	85.3	1.62 (1.37–1.91)	1.48 (1.09–2.03)	1.51 (1.10–2.08)	1.13 (0.75–1.71)	1.50 (1.10–2.05)	1.12 (0.74–1.70)
**Socio-demographic characteristics**								
Sex								
	Male	60.1	1.41 (1.14–1.75)	0.92 (0.69–1.22)	0.92 (0.68–1.25)	-	-	-
	Female	67.2	1.50 (1.24–1.83)	1 (Ref)	1 (Ref)	-	-	-
Age								
	≤34	54.9	1.82 (1.50–2.21)	1 (Ref)	1 (Ref)	-	-	1 (Ref)
	35–44	38.5	1.19 (0.89–1.59)	0.64 (0.45–0.90)	0.64 (0.45–0.91)	-	-	0.69 (0.48–1.00)
	45–54	21.3	1.17 (0.79–1.74)	0.61 (0.40–0.94)	0.65 (0.41–1.03)	-	-	0.62 (0.40–0.97)
	≥55	12.8	1.33 (0.83–2.14)	0.74 (0.45–1.22)	0.73 (0.42–1.25)	-	-	0.82 (0.49–1.37)
Marital Status								
	Married	77.5	1.46 (1.21–1.75)	1 (Ref)	1 (Ref)	-	-	-
	Single	16.2	1.36 (0.89–2.06)	0.87 (0.55–1.37)	0.76 (0.47–1.21)	-	-	-
	Widowed	20.0	1.05 (0.69–1.61)	0.71 (0.44–1.13)	0.80 (0.48–1.32)	-	-	-
	Divorced	10.7	2.53 (1.73–3.69)	1.66 (1.09–2.52)	1.66 (1.09–2.53)	-	-	-
	Missing	3.2	1.58 (0.66–3.80)	1.03 (0.41–2.57)	0.81 (0.29–2.23)	-	-	-
Highest level of education								
	None or Primary	29.7	1.58 (1.19–2.11)	1 (Ref)	1 (Ref)	-	-	-
	Secondary or Tertiary	63.3	1.53 (1.25–1.86)	0.98 (0.69–1.38)	0.92 (0.64–1.32)	-	-	-
	Missing	34.5	1.27 (0.95–1.71)	0.80 (0.53–1.21)	0.78 (0.52–1.19)	-	-	-
**Clinical Characteristics**								
Baseline CD4 Count Recorded								
	No	77.1	1.71 (1.44–2.03)	1 (Ref)	-	1 (Ref)	-	1 (Ref)
	Yes	50.5	1.11 (0.85–1.44)	0.63 (0.46–0.85)	-	0.87 (0.56–1.36)	-	0.88 (0.56–1.38)
CD4 count at ART Initiation								
	0–200	22.7	1.23 (0.85–1.78)	1 (Ref)	-	1 (Ref)	-	1 (Ref)
	201–350	13.0	0.77 (0.41–1.43)	0.64 (0.32–1.28)	-	0.62 (0.31–1.27)	-	0.58 (0.29–1.19)
	351–500	8.1	1.35 (0.75–2.45)	1.17 (0.61–2.24)	-	1.14 (0.57–2.29)	-	1.00 (0.50–2.04)
	≥501	6.6	1.06 (0.51–2.23)	0.89 (0.39–2.02)	-	0.70 (0.30–1.65)	-	0.72 (0.32–1.65)
	Missing	77.1	1.71 (1.44–2.03)	1.47 (0.99–2.19)	-	-	-	-
WHO Clinical Stage at ART Initiation								
	I	40.0	1.88 (1.50–2.35)	1 (Ref)	-	1 (Ref)	-	-
	II	46.8	1.20 (0.92–1.55)	0.60 (0.43–0.84)	-	0.67 (0.47–0.97)	-	-
	III or IV	37.2	1.29 (0.97–1.71)	0.60 (0.42–0.86)	-	0.74 (0.50–1.10)	-	-
	Missing	3.6	2.51 (1.31–4.83)	1.18 (0.57–2.41)	-	1.40 (0.68–2.88)	-	-
Days between HIV diagnosis and ART initiation								
	Same day	22.4	2.18 (1.65–2.89)	1 (Ref)	-	1 (Ref)	-	1 (Ref)
	1–7	32.8	1.53 (1.16–2.01)	0.65 (0.44–0.96)	-	0.70 (0.47–1.05)	-	0.73 (0.49–1.10)
	8–28	36.4	1.32 (0.99–1.75)	0.56 (0.38–0.83)	-	0.69 (0.45–1.05)		0.66 (0.43–0.99)
	>28	27.9	1.18 (0.84–1.67)	0.52 (0.34–0.79)	-	0.70 (0.44–1.11)		0.63 (0.40–0.99)
**Health Facility Characteristics**								
Health Facility Management								
	Central Government	80.1	1.51 (1.26–1.81)	1 (Ref)	-	-	1 (Ref)	-
	Rural District Council	25.2	1.43 (1.03–1.98)	0.88 (0.61–1.25)	-	-	0.90 (0.56–1.46)	-
	Faith-based Mission	22.3	1.39 (0.98–1.98)	0.85 (0.57–1.25)			0.78 (0.51–1.20)	-
Study District								
	Mutasa	39.4	1.60 (1.25–2.05)	1 (Ref)		-	1 (Ref)	-
	Makoni	88.1	1.42 (1.19–1.69)	0.92 (0.69–1.22)	-	-	0.80 (0.59–1.10)	-
Health Facility Type								
	Hospitals	86.6	1.49 (1.25–1.77)	1 (Ref)	-	-	1 (Ref)	-
	Large Health Centre	28.0	1.50 (1.11–2.03)	0.99 (0.71–1.38)	-	-	0.96 (0.61–1.51)	-
	Small Clinic	13.0	1.31 (0.81–2.10)	0.84 (0.53–1.33)	-	-	0.79 (0.45–1.40)	-

^1^ Person days per 1000.

^2^ Adjusted for enrolment period and socio-demographic characteristics.

^3^ Adjusted for enrolment period and clinical characteristics.

^4^ Adjusted for enrolment period and health facility characteristics.

^5^ Adjusted for enrolment period and all covariates p<0.2 in bivariate analysis.

Attrition levels were approximately 40% lower in patients aged 35–54 years at enrolment compared to those aged ≤34 years. Incidence rates and sub-hazard ratios for attrition did not differ greatly by health facility characteristics; indicating that levels of attrition at primary care facilities–a key component of decentralised health service delivery–were not higher than those at first-referral facilities. Correlates of attrition were similar in a sensitivity analysis where all absences >90 days were included (S2 Table).

S4 Table shows sub-hazard ratios of attrition by socio-demographic, clinical and health facility characteristic for each study time period individually. No associations were found in the pre- treat-all time period. Younger age, divorced status (*versus* married), CD4 count ART initiation, and ART initiation on the same day as HIV diagnosis had statistically significant associations with attrition in the treat-all period.

## 4 Discussion

In cohorts of patients in Manicaland, Zimbabwe, retention in care increased from 54.2% to 59.0% following the introduction of national policies recommending ART for all PLHIV. All-cause mortality declined but attrition from care increased in the treat-all period compared with the pre-treat-all period. Modelled sub-hazard ratios indicate an increase in AFC amongst those accessing care during the treat-all period. The strength of this association, however, was reduced when accounting for age and clinical characteristics such as the availability of a baseline CD4 count, WHO clinical stage, and ART initiation on the same day as HIV diagnosis.

These findings provide evidence that, as was intended, the introduction and implementation of treat-all guidelines in east Zimbabwe resulted in earlier and more rapid ART initiation. This was achieved largely by eliminating the need to establish eligibility for ART, previously done through CD4 cell count monitoring which declined in line with findings reported in other settings [17, 19]. However, these developments also led to changes in the patient population (Table 1) who became younger, less likely to have experienced an HIV-related illness (WHO stage I) prior to initiating treatment, and less well prepared for initiation on lifelong ART. Our findings indicate that these factors may serve as important mediators in explaining the increased occurrence of attrition in the treat-all era [31]. If this is the case, interventions to reduce ART attrition could include differentiated pre-ART counselling and intensified post-ART support for individuals in groups at high risk of attrition (e.g. young PLHIV, those who receive rapid ART initiation, and those with a high CD4 cell count or in WHO stage I or II at ART initiation), and increased access to clinical monitoring. Targeted and continuous face-to-face and telephone-based counselling, and the use of mobile phone messaging services which have been previously reported to improve rates of adherence to ART and retention in care, may be particularly useful [32, 33]. Access to clinical monitoring could be increased though use of point-of-care technologies for CD4 testing and improved sample transportation in locations where HIV care is decentralised [31].

Few studies have reported on patient outcomes in the treat-all era; however, our findings are consistent with those reported by others in sub-Saharan Africa. For example, younger age was found to be associated with increased risk of attrition in Malawi, Ethiopia and South Africa [12, 34, 35] and in Mozambique, attrition was associated with lack of regular CD4 count amongst community ART groups [36]. To the best of our knowledge, only one other study has reported on the incidence and determinants of attrition from HIV care in Zimbabwe since treat-all recommendations were introduced. Makurumidze and colleagues reported a 73% increased hazard of attrition in the treat-all period, amongst patients within nine study districts selected to pilot the treat-all programme in Zimbabwe, including Mutasa and Makoni districts included in the present analysis [20]. In this analysis however, researchers used a 180 day interval since the last clinic visit to determine AFC, in contrast to the 90 day used in the present study, and death was not considered to be a competing event [20]. In addition, data collected in the Makurumidze study were limited to health facilities with electronic patient monitoring systems (ePMS) in the HIV programme, a system that was primarily used at high-volume sites in the pre- treat-all era [37]. Despite these differences, results reported were congruent with our findings of an increase in same-day ART initiation in the treat-all period, and higher attrition amongst younger age groups, and those asymptomatic at the time of ART initiation (WHO stage I).

The question of how quickly ART should be initiated following HIV diagnosis and enrolment into care has been common as national programmes have adapted to treat-all [38]. In our analysis, we observed a significant rise in the proportion of patients initiating ART on the same day as HIV diagnosis or within 7 days (Table 1). The synchronous association between same-day ART initiation and AFC suggests that in the context of Zimbabwe–as in South Africa—rapid ART initiation may be a cause for concern [21, 39]. In other settings, however, conflicting results have been reported, primarily within randomised trials or amongst pregnant women where same-day initiation was associated with improved outcomes [38, 40, 41]. Further research is required to better understand ART initiation dynamics and the effect that this may have on attrition in the longer term.

While common themes have emerged, differences in AFC across studies may occur due to heterogeneity of study settings, as well as variations in defining attrition. Recently, Chi and colleagues from the IeDEA collaboration (International Epidemiological Databases to Evaluate AIDS) recommended a threshold of ≥180 days since the last clinic visit as being the threshold with the lowest misclassification rate [42]. In Zambia, the best-performing definition of AFC was found to be 56 days after a missed visit while numerous other studies, including two studies in Zimbabwe, have used a threshold of 3 months or 90 days since the last clinic appointment [43]. In the era of differentiated care, it may be difficult to ascertain an optimum threshold for AFC as time between clinic visits can be lengthened for patients considered to be stable on ART, potentially leading to false measurements of attrition or interruptions from care.

This analysis has a number of limitations that should be noted. First, the purposive selection of health facilities into the study may limit the generalizability of the findings, particularly given the predominance of rural health facilities in the included study districts, and the exclusion of urban areas in Manicaland province, such as Mutare. In addition, health facilities had differing patient population sizes due to the inclusion of clinics at various levels of decentralisation. Second, while differences between the pre- treat all, and treat-all time periods have been observed in these data, it cannot be inferred that these are directly due to the change in national policies as opposed to other confounding or unobserved factors. These include religious beliefs, particularly amongst those belonging to organizations that promote faith-healing and alternative medicines [44, 45], economic changes, differentiated care, and co-morbidities, among others. Third, data were collected in the early implementation phase of the treat-all approach when there were some clients previously enrolled into care and already eligible for ART under the new guidelines. While this is a strength of this analysis, given the early evidence reported here, programme delivery may have changed subsequently, warranting further research to confirm the associations found. Data were collected amongst patients newly enrolled into care, and initiated onto ART. Patients who may have experienced attrition before ART initiation are thus excluded which may underestimate attrition from ART care overall.

## 5 Conclusion

Incidence of attrition increased following the introduction of treat-all policies for HIV in east Zimbabwe. Increases in attrition were driven largely by initiation of ART on the same-day as HIV diagnosis, which rose in the treat all time period. This is a concern as the success of national HIV treatment programmes is reliant upon the continued retention of PLHIV in clinical care. Interventions such as differentiated adherence counselling for patient groups at increased risk of attrition, improved access to clinical monitoring, and use of peer support programmes to improve retention may be beneficial as treat-all implementation continues.

## Supporting information

S1 Table Characteristics of twelve health facilities from which the patient population were drawn. (DOCX)

S2 Table Crude and adjusted measures of association between hypothesized predictors and attrition from care, for all absences >90 days. (DOCX)

S3 Table Crude measures of association between clinical characteristics at ART initiation and study period of enrolment. (DOCX)

S4 Table Crude and adjusted measures of association between hypothesized predictors and attrition, stratified by study period. (DOCX)
