# Supplementary material for: Incidence and predictors of attrition among patients receiving ART in eastern Zimbabwe before, and after the introduction of universal ‘treat-all’ policies: A competing risk analysis
Source: PLOS Glob Public Health. 2021 Oct 13;1(10):e0000006. doi: 10.1371/journal.pgph.0000006 (PMC10021537; doi:10.1371/journal.pgph.0000006)
Supplement: S1 Table — (DOCX) [file pgph.0000006.s001.docx]

**S1 Table:** Characteristics of twelve health facilities from which the patient population were drawn.

|  | | **Hospitals^1^**  **(N=4)** | **Large Health Centres**  **(N=4)** | **Small Clinics**  **(N=4)** | **Total**  **(N=12)** |
| --- | --- | --- | --- | --- | --- |
| Patient Volume^2,3^ | | 814.5 (751) 510.5 | 413.8 (194.2) 353.5 | 194.8 (93.5) 161 | 474.3 (488.2) 324 |
| Number of beds^2^ | | 97.8 (110.3) 59 | 7.25 (4.92) 7 | 5.25 (3.86) 3.5 | 36.8 (73.2) 11 |
| Human Resources^2^ | |  |  |  |  |
|  | Medical Doctors | 2.5 (2.65) 2 | 0 (0) 0 | 0.25 (0.5) 0 | 0.92 (1.83) 0 |
|  | Registered Nurses and Midwives | 40 (53.6) 20 | 0.75 (0.5) 1 | 0.5 (0.58) 0.5 | 13.8 (34.0) 1 |
|  | Laboratory Staff | 1.25 (2.5) 0 | 0 (0) 0 | 0 (0) 0 | 0.42 (1.44) 0 |
|  | Community outreach workers | 27 22.7 () 27.5 | 8 (5.89) 9 | 12.3 (9.3) 8.5 | 15.8 (15.7) 10 |
|  | Health-care staff per 100 patients | 13.7 (5.86) 12.6 | 3.62 (1.75) 4.39 | 8.29 (1.34) 8.31 | 8.54 (5.41) 7.73 |
| Laboratory Capacity on site | |  |  |  |  |
|  | CD4 Count | 3 (75.0%) | 0 (0%) | 0 (0%) | 3 (25.0%) |
|  | Viral Load | 0 (0%) | 0 (0%) | 0 (0%) | 0 (0%) |
| Financial Resources and Support | |  |  |  |  |
|  | Results Based Funding Support | 2 (50.0%) | 4 (100%) | 4 (100%) | 10 (83.3%) |
|  | Payment Required for HTC or ART | 1 (25.0%) | 0 (0%) | 0 (0%) | 1 (8.3%) |
| HTC Service Delivery | |  |  |  |  |
|  | HIV tests conducted over previous three months^2^ | 2052.5 (2075.9) 1323 | 299.3 (112.6) 305.5 | 267.3 (288.3) 131 | 873 (1400.2) 390.5 |
|  | At least one stock-out of HIV test kits in previous year^4^ | 0 (0%) | 1 (25.0%) | 1 (25.0%) | 2 (16.7%) |

^1^ First-referral level facilities - district and rural hospitals

^2^ Summary Statistics: Mean (SD) Median

^3^ Total number receiving 1st or 2nd line ART

^4^ Stock-outs in previous year
