# Supplementary material for: Incidence and predictors of attrition among patients receiving ART in eastern Zimbabwe before, and after the introduction of universal ‘treat-all’ policies: A competing risk analysis
Source: PLOS Glob Public Health. 2021 Oct 13;1(10):e0000006. doi: 10.1371/journal.pgph.0000006 (PMC10021537; doi:10.1371/journal.pgph.0000006)
Supplement: S2 Table — (DOCX) [file pgph.0000006.s002.docx]

**S2 Table. Crude and adjusted measures of association between hypothesized predictors and attrition from care, for all absences >90 days.**

|  |  |  |  | **Bivariate Analysis** | **Multivariable Model 1^2^** | **Multivariable Model 2^3^** | **Multivariable Model 3^4^** | **Multivariable Model 4^5^** |
| --- | --- | --- | --- | --- | --- | --- | --- | --- |
|  |  | **Person-days^1^** | **Incidence Rate (95% CI)** | **cSHR (95% CI)** | **aSHR (95% CI)** | **aSHR (95% CI)** | **aSHR (95% CI)** | **aSHR (95% CI)** |
| Enrolment Time Period | |  |  |  |  |  |  |  |
|  | Pre- Treat-All | 39.2 | 1.84 (1.46 - 2.31) | 1 (Ref) | 1 (Ref) | 1 (Ref) | 1 (Ref) | 1 (Ref) |
|  | Treat-All | 72.8 | 3.46 (3.06 - 3.92) | 2.20 (1.70 - 2.87) | 2.29 (1.75 - 2.99) | 2.13 (1.56 - 2.91) | 2.26 (1.73 - 2.93) | 2.00 (1.47 - 2.74) |
| **Socio-demographic characteristics** | | | | | | | | |
| Sex | |  |  |  |  |  |  |  |
|  | Male | 52.8 | 2.86 (2.44 - 3.35) | 0.96 (0.78 - 1.19) | 0.93 (0.74 - 1.16) | - | - | - |
|  | Female | 58.9 | 2.91 (2.50 - 3.38) | 1 (Ref) | 1 (Ref) | - | - | - |
| Age | |  |  |  |  |  |  |  |
|  | ≤34 | 48.9 | 3.13 (2.67 - 3.67) | 1 (Ref) | 1 (Ref) | - | - | - |
|  | 35-44 | 33.4 | 2.70 (2.19 - 3.32) | 0.84 (0.65 - 1.09) | 0.83 (0.64 - 1.07) | - | - | - |
|  | 45-54 | 18.8 | 2.71 (2.06 - 3.57) | 0.82 (0.61 - 1.12) | 0.89 (0.65 - 1.21) | - | - | - |
|  | ≥55 | 10.9 | 2.74 (1.92 - 3.92) | 0.90 (0.62 - 1.31) | 0.92 (0.61 - 1.38) | - | - | - |
| Marital Status | |  |  |  |  |  |  |  |
|  | Married | 68.4 | 2.78 (2.41 - 3.20) | 1 (Ref) | 1 (Ref) | - | - | - |
|  | Single | 13.8 | 3.13 (2.32 - 4.22) | 1.06 (0.77 - 1.47) | 0.93 (0.66 - 1.31) | - | - | - |
|  | Widowed | 17.6 | 2.56 (1.91 - 3.43) | 0.91 (0.66 - 1.28) | 1.00 (0.70 - 1.44) | - | - | - |
|  | Divorced | 9.5 | 3.90 (2.83 - 5.38) | 1.39 (0.97 - 1.98) | 1.45 (1.03 - 2.04) | - | - | - |
|  | Missing | 2.7 | 3.33 (1.73 - 6.39) | 1.14 (0.59 - 2.20) | 0.91 (0.46 - 1.78) | - | - | - |
| Highest level of education | | | | | | | | |
|  | None or Primary | 26.7 | 2.81 (2.24 - 3.52) | 1 (Ref) | 1 (Ref) | - | - | - |
|  | Secondary or Tertiary | 55.6 | 2.91 (2.50 - 3.40) | 1.06 (0.82 - 1.37) | 1.07 (0.82 - 1.41) | - | - | - |
|  | Missing | 29.6 | 2.94 (2.38 - 3.62) | 1.06 (0.78 - 1.43) | 1.08 (0.80 - 1.47) | - | - | - |
| **Clinical Characteristics** | | | | | | | | |
| Baseline CD4 Count Recorded | | | | | | | | |
|  | No | 66.9 | 3.38 (2.97 - 3.85) | 1 (Ref) | - | 1 (Ref) | - | 1 (Ref) |
|  | Yes | 45.1 | 2.17 (1.78 - 2.65) | 0.59 (0.47 - 0.75) | - | 1.02 (0.72 - 1.46) | - | 0.98 (0.68 - 1.40) |
| CD4 count at ART Initiation | | | | | | | | |
|  | 0-200 | 20.2 | 2.33 (1.75 - 3.10) | 1 (Ref) | - | 1 (Ref) | - | 1 (Ref) |
|  | 201-350 | 11.8 | 1.87 (1.23 - 2.84) | 0.84 (0.51 - 1.38) | - | 0.86 (0.52 - 1.45) | - | 0.89 (0.53 - 1.48) |
|  | 351-500 | 7.4 | 2.31 (1.44 - 3.71) | 1.05 (0.62 - 1.77) | - | 1.28 (0.77 - 2.14) | - | 1.24 (0.74 - 2.08) |
|  | ≥501 | 5.8 | 2.08 (1.18 - 3.66) | 0.92 (0.48 - 1.77) | - | 0.70 (0.25 - 1.40) | - | 0.68 (0.34 - 1.38) |
|  | Missing | 66.9 | 3.38 (2.97 - 3.85) | 1.61 (1.18 - 2.20) | - | - | - | - |
| WHO Clinical Stage at ART Initiation | | | | | | | | |
|  | I | 35.3 | 3.35 (2.79 - 4.01) | 1 (Ref) | - | 1 (Ref) | - | - |
|  | II | 40.9 | 2.62 (2.16 - 3.16) | 0.71 (0.56 - 0.92) | - | 0.90 (0.69 - 1.18) | - | - |
|  | III or IV | 33.3 | 2.46 (1.98 - 3.06) | 0.61 (0.46 - 0.81) | - | 0.92 (0.69 - 1.24) | - | - |
|  | Missing | 2.5 | 6.73 (4.19 - 10.8) | 2.10 (1.30 - 3.37) | - | 2.73 (1.61 - 4.62) | - | - |
| ART initiated on same day as HIV diagnosis | | | | | | | | |
|  | No | 86.5 | 2.59 (2.27 - 2.95) | 1 (Ref) | - | 1 (Ref) | - | 1 (Ref) |
|  | Yes | 18.8 | 4.20 (3.37 - 5.23) | 1.83 (1.43 - 2.35) | - | 1.48 (1.13 - 1.95) | - | 1.69 (1.27 - 2.23) |
| **Health Facility Characteristics** | | | | | | | | |
| Health Facility Management | | | | | | | | |
|  | Central Government | 70.7 | 2.84 (2.47 - 3.26) | 1 (Ref) | - | - | 1 (Ref) | - |
|  | Rural District Council | 23.3 | 2.57 (2.00 - 3.31) | 0.83 (0.63 - 1.10) | - | - | 0.78 (0.54 - 1.12) | - |
|  | Faith-based Mission | 17.9 | 3.52 (2.75 - 4.50) | 1.15 (0.88 - 1.51) |  |  | 1.10 (0.82 - 1.48) | - |
| Study District | |  |  |  |  |  |  |  |
|  | Mutasa | 33.7 | 3.21 (2.66 - 3.88) | 1 (Ref) |  | - | 1 (Ref) | - |
|  | Makoni | 78.3 | 2.76 (2.41 - 3.15) | 0.88 (0.71 - 1.10) | - | - | 0.81 (0.64 - 1.03) | - |
| Health Facility Type | |  |  |  |  |  |  |  |
|  | Hospitals | 74.8 | 3.01 (2.64 - 3.43) | 1 (Ref) | - | - | 1 (Ref) | 1 (Ref) |
|  | Large Health Centre | 25.4 | 2.87 (2.29 - 3.62) | 0.94 (0.73 - 1.21) | - | - | 1.10 (0.78 - 1.55) | 0.80 (0.61 - 1.05) |
|  | Small Clinic | 11.8 | 2.20 (1.50 - 3.23) | 0.68 (0.47 - 0.98) | - | - | 0.78 (0.50 - 1.20) | 0.71 (0.46 - 1.09) |

^1^ Person days per 1000

^2^ Adjusted for enrolment period and socio-demographic characteristics

^3^ Adjusted for enrolment period and clinical characteristics

^4^ Adjusted for enrolment period and health facility characteristics

^5^ Adjusted for enrolment period and all covariates p<0.2 in bivariate analysis
