# Supplementary material for: Incidence and predictors of attrition among patients receiving ART in eastern Zimbabwe before, and after the introduction of universal ‘treat-all’ policies: A competing risk analysis
Source: PLOS Glob Public Health. 2021 Oct 13;1(10):e0000006. doi: 10.1371/journal.pgph.0000006 (PMC10021537; doi:10.1371/journal.pgph.0000006)
Supplement: S3 Table — (DOCX) [file pgph.0000006.s003.docx]

**S3 Table. Crude measures of association between clinical characteristics at ART initiation and study period of enrolment.**

|  |  | No. (%)^1^ | cOR (95%CI) |
| --- | --- | --- | --- |
| Baseline CD4 count recorded | |  |  |
|  | No | 381 (87.2%) | 1 (Ref) |
|  | Yes | 94 (37.0%) | 0.09 (0.06 - 0.13) |
| CD4 count at ART Initiation | |  |  |
|  | 0-200 | 42 (35.0%) | 1 (Ref) |
|  | 201-350 | 24 (38.1%) | 1.14 (0.61 - 2.15) |
|  | 351-500 | 10 (25.0%) | 0.62 (0.28 - 1.39) |
|  | ≥501 | 18 (58.1%) | 2.57 (1.15 - 5.76) |
|  | Missing | 381 (87.2%) | 12.6 (7.91 - 20.2) |
| WHO Clinical Stage at ART Initiation | |  |  |
|  | I | 198 (87.6%) | 1 (Ref) |
|  | II | 156 (65.0%) | 0.26 (0.16 - 0.42) |
|  | III or IV | 111 (54.7%) | 0.17 (0.11 - 0.28) |
|  | Missing | 10 (45.5%) | 0.12 (0.05 - 0.30) |
| ART initiated on same day as HIV diagnosis | |  |  |
|  | No | 333 (64.4%) | 1 (Ref) |
|  | Yes | 112 (85.5%) | 3.26 (1.94 - 5.47) |

^1^Proportion of patients enrolled during the treat-all period
