# Supplementary material for: Incidence and predictors of attrition among patients receiving ART in eastern Zimbabwe before, and after the introduction of universal ‘treat-all’ policies: A competing risk analysis
Source: PLOS Glob Public Health. 2021 Oct 13;1(10):e0000006. doi: 10.1371/journal.pgph.0000006 (PMC10021537; doi:10.1371/journal.pgph.0000006)
Supplement: S4 Table — (DOCX) [file pgph.0000006.s004.docx]

**S4 Table. Crude and adjusted measures of association between hypothesized predictors and attrition, stratified by study period.**

|  |  | **Pre-Treat All** | | | **Treat All** | | |
| --- | --- | --- | --- | --- | --- | --- | --- |
|  |  |  | **Bivariate Model** | **Multivariable Model^1^** |  | **Bivariate Model** | **Multivariable Model^1^** |
|  |  | **IR (95% CI)** | **cSHR (95% CI)** | **aSHR (95% CI)** | **IR (95% CI)** | **cSHR (95% CI)** | **aSHR (95% CI)** |
| Sex | |  |  |  |  |  |  |
|  | Male | 0.98 (0.91 - 1.84) | 0.76 (0.43 - 1.33) | - | 1.60 (1.26 - 2.03) | 0.96 (0.69 - 1.33) | - |
|  | Female | 1.30 (0.62 - 1.55) | 1 (Ref) | - | 1.62 (1.28 - 2.05) | 1 (Ref) | - |
| Age | |  |  |  |  |  |  |
|  | ≤34 | 1.35 (0.91 - 2.02) | 1 (Ref) | - | 2.04 (1.63 - 2.56) | 1 (Ref) | 1 (Ref) |
|  | 35-44 | 1.90 (0.49 - 1.61) | 0.67 (0.33 - 1.39) | - | 1.33 (0.96 - 1.86) | 0.62 (0.42 - 0.92) | 0.69 (0.45 - 1.04) |
|  | 45-54 | 0.97 (0.48 - 1.93) | 0.67 (0.31 - 1.44) | - | 1.30 (0.81 - 2.08) | 0.61 (0.37 - 1.02) | 0.63 (0.37 - 1.08) |
|  | ≥55 | 1.74 (0.73 - 3.65) | 1.40 (0.62 - 3.17) | - | 1.14 (0.61 - 2.12) | 0.55 (0.29 - 1.05) | 0.59 (0.31 - 1.11) |
| Marital Status | |  |  |  |  |  |  |
|  | Married | 1.28 (0.91 - 1.80) | 1 (Ref) | - | 1.55 (1.24 - 1.92) | 1 (Ref) | - |
|  | Single | 0.63 (0.20 - 1.94) | 0.47 (0.14 - 1.59) | - | 1.65 (1.05 - 2.59) | 0.99 (0.61 - 1.61) | - |
|  | Widowed | 0.96 (0.48 - 1.91) | 0.71 (0.33 - 1.52) | - | 1.11 (0.64 - 1.91) | 0.71 (0.39 - 1.30) | - |
|  | Divorced | 1.62 (0.67 - 3.88) | 1.03 (0.40 - 2.67) | - | 2.90 (1.91 - 4.41) | 1.93 (1.21 - 3.07) | - |
|  | Missing | 2.52 (0.25 - 17.9) | 1.09 (0.12 - 9.74) |  | 1.45 (0.54 - 3.86) | 0.97 (0.35 - 2.64) |  |
| Highest level of education | |  |  |  |  |  |  |
|  | None or Primary | 1.32 (0.75 - 2.33) | 1 (Ref) | 1 (Ref) | 1.70 (1.22 - 2.37) | 1 (Ref) | - |
|  | Secondary or Tertiary | 1.43 (1.01 - 2.04) | 1.15 (0.60 - 2.22) | 1.08 (0.54 - 2.17) | 1.58 (1.24 - 2.01) | 0.92 (0.62 - 1.38) | - |
|  | Missing | 0.60 (0.28 - 1.25) | 0.47 (0.18 - 1.19) | 0.60 (0.23 - 1.54) | 1.61 (1.17 - 2.23) | 0.93 (0.59 - 1.46) | - |
| Baseline CD4 Count Recorded | | |  |  |  |  |  |
|  | No | 1.49 (0.90 - 2.48) | 1 (Ref) | - | 1.74 (1.45 - 2.09) | 1 (Ref) | 1 (Ref) |
|  | Yes | 1.08 (0.78 - 1.51) | 0.82 (0.45 - 1.49) | - | 1.15 (0.75 - 1.77) | 0.64 (0.41 - 1.00) | 0.71 (0.35 - 1.43) |
| CD4 count at ART Initiation | |  |  |  |  |  |  |
|  | 0-200 | 1.31 (0.84 - 2.03) | 1 (Ref) | - | 1.08 (0.54 - 2.15) | 1 (Ref) | 1 (Ref) |
|  | 201-350 | 0.84 (0.40 - 1.76) | 0.68 (0.30 - 1.53) | - | 0.63 (0.20 - 1.95) | 0.59 (0.16 - 2.22) | 0.51 (0.13 - 1.93) |
|  | 351-500 | 0.86 (0.36 - 2.06) | 0.70 (0.27 - 2.85) | - | 2.61 (1.17 - 5.80) | 2.54 (1.04 - 6.21) | 2.35 (0.84 - 6.57) |
|  | ≥501 | 1.04 (0.43 - 3.24) | 0.78 (0.24 - 2.51) | - | 1.08 (0.41 - 2.88) | 1.07 (0.32 - 3.56) | 1.03 (0.30 - 3.47) |
|  | Missing | 1.49 (0.90 - 2.48) | 1.04 (0.53 - 2.02) | - | 1.74 (1.45 - 2.09) | 1.71 (0.87 - 3.39) | - |
| WHO Clinical Stage at ART Initiation | | |  |  |  |  |  |
|  | I | 1.36 (0.65 - 2.85) | 1 (Ref) | - | 1.95 (1.54 - 2.48) | 1 (Ref) | - |
|  | II | 1.08 (0.69 - 1.70) | 0.70 (0.31 - 1.59) | - | 1.26 (0.92 - 1.75) | 0.63 (0.43 - 0.92) | - |
|  | III or IV | 1.09 (0.70 - 1.71) | 0.65 (0.28 - 1.51) | - | 1.46 (1.02 - 2.10) | 0.68 (0.44 - 1.04) | - |
|  | Missing | 2.21 (0.92 - 5.31) | 1.23 (0.36 - 4.17) | - | 2.85 (1.07 - 7.59) | 1.60 (0.66 - 3.92) | - |
| ART initiated on same day as HIV diagnosis | | |  |  |  |  |  |
|  | No | 1.14 (0.84 - 1.54) | 1 (Ref) | 1 (Ref) | 1.47 (1.20 - 1.81) | 1 (Ref) | 1 (Ref) |
|  | Yes | 2.24 (1.12 - 4.47) | 1.99 (0.88 - 4.50) | 1.80 (0.77 - 4.19) | 2.18 (1.60 - 3.00) | 1.53 (1.07 - 2.19) | 1.40 (0.97 - 2.01) |
| **Health Facility Characteristics** | | | | | | | |
| Health Facility Management | |  |  |  |  |  |  |
|  | Central Government | 1.29 (0.92 - 1.80) | 1 (Ref) | - | 1.62 (1.31 - 2.00) | 1 (Ref) | - |
|  | Rural District Council | 0.68 (0.28 - 1.64) | 0.42 (0.17 - 1.10) | - | 1.72 (1.21 - 2.44) | 1.02 (0.69 - 1.50) | - |
|  | Faith-based Mission | 1.28 (0.71 - 2.61) | 0.85 (0.43 - 1.70) | - | 1.47 (0.95 - 2.27) | 0.85 (0.53 - 1.37) | - |
| Study District | |  |  |  |  |  |  |
|  | Mutasa | 1.50 (1.00 - 2.23) | 1 (Ref) | - | 1.66 (1.21 - 2.27) | 1 (Ref) | - |
|  | Makoni | 0.99 (0.67 - 1.45) | 0.70 (0.41 - 1.19) | - | 1.60 (1.31 - 1.95) | 0.98 (0.69 - 1.39) | - |
| Health Facility Type | |  |  |  |  |  |  |
|  | Hospital | 1.25 (0.90 - 1.73) | 1 (Ref) | - | 1.61 (1.32 - 1.97) | 1 (Ref) | - |
|  | Large Health Centre | 0.89 (0.42 - 1.86) | 0.63 (0.28 - 1.39) | - | 1.73 (1.24 - 2.40) | 1.08 (0.74 - 1.57) | - |
|  | Small Clinic | 1.24 (0.59 - 2.60) | 1.09 (0.51 - 2.26) | - | 1.36 (0.73 - 2.53) | 0.75 (0.42 - 1.33) | - |

^1^ Adjusted for all covariates p<0.2 in bivariate analysis
